# Contours of a causal feedback mechanism between adaptive personality and psychosocial function in patients with personality disorders: a secondary analysis from a randomized clinical trial

**DOI:** 10.1186/s12888-017-1365-4

**Published:** 2017-06-05

**Authors:** Ole Klungsøyr, Bjørnar Antonsen, Theresa Wilberg

**Affiliations:** 1 Oslo Centre for Biostatistics and Epidemiology, Section for treatment research, Department for Research and Education, Division of Mental Health and Addiction, Oslo University Hospital, PO Box 4959, Nydalen, 0424 Oslo, Norway; 2 Department for Personality Psychiatry, Division of Mental Health and Addiction, Oslo University Hospital, Oslo, Norway; 3 Institute for Clinical Medicine, University of Oslo, Oslo, Norway; 4 Section for treatment research, Department for Research and Education, Division for Mental Health and Addiction, Oslo University Hospital, Oslo, Norway

**Keywords:** Marginal structural model, Personality disorders, Psychosocial function, Personality functioning

## Abstract

**Background:**

Patients with personality disorders commonly exhibit impairment in psychosocial function that persists over time even with diagnostic remission. Further causal knowledge may help to identify and assess factors with a potential to alleviate this impairment. Psychosocial function is associated with personality functioning which describes personality disorder severity in DSM-5 (section III) and which can reportedly be improved by therapy.

**Methods:**

The reciprocal association between personality functioning and psychosocial function was assessed, in 113 patients with different personality disorders, in a secondary longitudinal analysis of data from a randomized clinical trial, over six years. Personality functioning was represented by three domains of the Severity Indices of Personality Problems: Relational Capacity, Identity Integration, and Self-control. Psychosocial function was measured by Global Assessment of Functioning. The marginal structural model was used for estimation of causal effects of the three personality functioning domains on psychosocial function, and vice versa. The attractiveness of this model lies in the ability to assess an effect of a time – varying exposure on an outcome, while adjusting for time – varying confounding.

**Results:**

Strong causal effects were found. A hypothetical intervention to increase Relational Capacity by one standard deviation, both at one and two time-points prior to assessment of psychosocial function, would increase psychosocial function by 3.5 standard deviations (95% CI: 2.0, 4.96). Significant effects of Identity Integration and Self-control on psychosocial function, and from psychosocial function on all three domains of personality functioning, although weaker, were also found.

**Conclusion:**

This study indicates that persistent impairment in psychosocial function can be addressed through a causal pathway of personality functioning, with interventions of at least 18 months duration.

## Background

Studies of coherent and structured treatments for patients with personality disorders (PDs) have reported promising results, improving the outlook for such patients, especially those with borderline PD [1]. However, while diagnostic remission and significant reduction of symptomatic distress are commonly reported, the benefits are less consistent with regards to psychosocial function, a key concept in PD diagnoses [2]. The few prospective studies with long-term follow-up demonstrate both persistent impairment and limited improvement in psychosocial function [3–6]. More knowledge is needed about causal effects on psychosocial function [7, 8].

The criteria for a PD diagnosis include psychosocial dysfunction, defined in DSM-5 as “an enduring pattern of inner experience and behavior that leads to clinically significant distress or impairment in social, occupational, or other important areas of functioning” [2]. Accordingly, impaired psychosocial function is associated with most measures of personality pathology [4, 5, 8–12]. However, this association is attenuated by low temporal stability in the PD criteria, reflected in studies with long follow-up, by high rates of diagnostic remission combined with long-term impairment in psychosocial function [5]. The DSM-IV criteria showed less predictive validity for psychosocial function than both nonadaptive personality traits (hypothesized to represent maladaptive personality functioning [13]) and the normal range, five-factor personality trait model (FFM) [9]. Previous studies indicate that normal range personality traits are marginally influenced by treatment [12, 14].

Personality (dys)functioning is an emerging construct describing PD severity in the DSM-5 alternative model (section III) [2] with a multi-domain model of personality [7]. It captures impairment levels in self and interpersonal relations and, despite differences, conceptually overlaps with personality traits [15, 16]. A relatively new research area focuses on changeable components of personality—more specifically (mal)adaptiveness—in order to assess treatment effects [17]. Such studies have revealed that therapy can improve (mal)adaptive personality functioning [18–20].

Personality functioning can be seen as one component of a broadly defined psychosocial function [7]. Such a view suggests that personality functioning has a causal influence on psychosocial function, which could potentially operate in both directions in a feedback mechanism, and of different strengths. A change in psychosocial function could consist of additive contributions across various components. Overlap in the definitions of personality functioning and psychosocial function makes a causal connection reasonable, although specific mechanisms are difficult to hypothesize. A crude label like “mental flexibility” could describe such a mechanism. Adaptiveness is a form of flexibility, and more flexibility is an obvious advantage in most areas of functioning. If adaptiveness can be improved by therapy, how much gain in psychosocial function can be achieved as a result of this improvement? Similarly, can more adaptive personality follow from improved psychosocial function? One example of an intervention directed at psychosocial function is vocational rehabilitation for persons with psychotic disorders [21]. Employment can be thought of as a potential contributor to improved self- and relational functioning. To assess the nature of a potential reciprocal association between personality functioning and psychosocial function, it is of interest to determine its magnitude, which direction is stronger, and the relevance of recent and more distant past levels. While it is established that PD criteria can predict psychosocial function, the reverse was not found [5]. Psychosocial function has also been predicted by maladaptive personality functioning [9], but not longitudinally in a reciprocal association, and not using an instrument sensitive to alterations in personality functioning [18]. An improved understanding of such a reciprocal association could inform the choice of interventions, such as therapy, vocational counseling, or a combination.

Causality and “causal inference” is a rapidly growing field in statistics, but is still somewhat controversial and object for heated debates across disciplines. Psychiatric research is no exception. Confronted with the causal influence in question in the present application, recognized experts have raised the following concern: “How can a construct have a causal influence on another if it is merely a component of the other?” In the causal inference methodology this is not a problem. One could easily ask the causal question of what would be the effect of one kilogram increase in body weight on the resulting BMI? Clearly body weight and BMI belong to the same construct, but one can perfectly assess the causal relationship between them, which in fact is deterministic. With respect to personality functioning and psychosocial function, the degree of overlap between them depends on how they are defined, which is not in focus here. The causal question of interest is based on clinical relevance alone. To identify and assess a new causal pathway to psychosocial function, which can be intervened on, would be important.

The marginal structural model (MSM) was developed in the statistical and epidemiological literature for the purpose of causal inference [22]. The paper that popularized the MSM in epidemiology has now been cited over 1800 times (Google Scholar, March 2016) [22], but rarely in psychology, with some notable recent exceptions [23, 24]. The MSM is particularly important when it is of interest to determine the effect of a time-varying variable (e.g., exposure) on an outcome (single or repeated measures), as is often the case in mental health applications. When the factors confounding the exposure–outcome association also vary with time and are affected by prior exposure, an ordinary regression (univariate or longitudinal model) will generally produce a biased estimate of the exposure effect, in many cases where the MSM is unbiased.

This study is a re-analysis of data from a randomized clinical trial. It is the first application of the MSM [25] to assess the reciprocal association between personality functioning (three domains) and psychosocial function, and the extent to which the effect of one on the other persists over time.

## Methods

### Sample

The present study sample is from the Ullevål Personality Project (UPP), a randomized study of patients with different PDs and outcomes of psychotherapeutic treatment given at different levels of care [6, 26, 27]. In UPP, patients were allocated either to outpatient individual psychotherapy (OIP) or to an intensive combination program (CP) comprising initial short-term day hospital treatment followed by long-term outpatient conjoint group and individual therapy. The therapists in both treatment conditions were mostly trained within a psychodynamically oriented psychotherapy tradition. Previous publications have described the treatments and therapists in detail [26, 28].

The study included 113 patients, of whom 75% were female. At baseline, the mean age was 31 years (SD = 7.3). The patients had an average of 4.4 years of education after junior secondary school, and 18% were married or cohabiting, 39% were living alone, and 33% were continuously medicated for the past 12 months. Randomized allocation led to 53% being in the CP group, and 47% in the OIP group [6]. Of the patients, 90% attended the 8-month follow-up, 80% attended the 18-month follow-up, 73% attended the 3-year follow-up, and 70% attended the 6-year follow-up.

The most frequent types of PDs were avoidant and borderline PD [26]. The low mean baseline level of psychosocial function indicated that this patient population generally suffered from severe PDs (see Results). Mean treatment length was 31 months (SD = 16 months) in the CP group, and 24 months (SD = 20 months) in the OIP group. At baseline, and at the 8-month, 18-month, 3-year, and 6-year follow-ups, the patients were evaluated using a wide range of measures, including assessments of psychosocial function and personality functioning. In the present analyses, the comparison of treatment conditions was not in focus. Instead, assigned treatment group was considered one of several potential confounders in the associations between personality functioning and psychosocial function.

### Measures

#### Psychosocial function

To measure psychosocial function, we applied Global Assessment of Functioning (GAF) [4, 13]. The GAF is a frequently used instrument that generates an observer-rated score ranging from 1 to 100, with a higher score indicating a higher level of functioning. The GAF score is generated by considering all available information regarding a subject’s psychiatric symptoms, and social and occupational functioning, and then determining a score in accordance with the lowest level of either the symptom or function realm [29]. The staff at the Department of Personality Psychiatry, Oslo University Hospital scored GAF at baseline, while the subsequent GAF interviews were carried out by research fellows. All raters were blinded to the treatment condition. Reliability was assessed by a comparison of these GAF scores and videotaped GAF interviews rated by independent experts (one at baseline, and the consensus score from two raters at 8, 18, 36 and 72 months). The GAF score reliability (intra-class correlation coefficient, ICC 2.1) was 0.56 at baseline, 0.81 at 8 months, 0.85 at 18 months, 0.94 at 3 years, and 0.92 at 6 years. Herein, we will not distinguish between psychosocial function and the GAF instrument that measures psychosocial function.

#### Personality functioning

The Severity Indices of Personality Problems (SIPP-118) is a self-report questionnaire that assesses the core components of (mal)adaptive personality functioning [17]. In the current study, we used a 60-item short version of SIPP that was specifically designed for research purposes (SIPP-118 SF). Of five domains, we selected the three with most explained variance in the original construction (64%): Identity Integration (IDENTITY), Self-control (SLFC), and Relational Capacity (REL) [17]. Each domain which is a linear combination of the 60 items, has a range from 1 (least adaptive) to 4 (most adaptive).

The SIPP-118 questionnaire covers a large part of the personality conceptualization from DSM-5, section III [30]. It was developed based on the notion that normal personality comprises constitutionally based temperaments, or basic tendencies [13, 31], as well as more adaptive capacities. The term adaptive capacities usually refers to the dynamic organization of personality concerning the regulation of self and relationships with others, and comprises characteristics that are believed to be changeable through therapy, such as affect and impulse regulation, identity, coping strategies, and acquired skills [20, 32]. Herein, no distinction will be made between personality functioning and the SIPP instrument used to measure personality functioning.

### Statistics

**Fig. 1:**
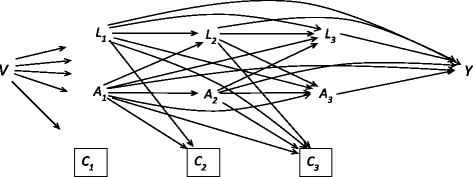
Causal graph (DAG [22]) of the study design, to illustrate the effect of exposure (A) on the outcome (Y). Symbols: baseline confounders (V), time – varying confounders including outcome (L) and censoring (C) (loss to follow-up), at baseline and follow-ups 1, 2, 3, in a Norwegian sample of 113 patients with personality disorders. An arrow symbolizes possible direct causal effect, the box around the C-symbol means “conditioned on” to reflect the fact that the analysis is restricted to those “not lost to follow-up”, a potential source of selection bias

The MSM is a model for the effect on an outcome, of hypothetical interventions on an exposure (or treatment) at one or more points in time. The resulting outcome of a hypothetical intervention is often referred to as a counterfactual outcome (the outcome had we, possibly contrary to fact, been able to change the exposure). With notation adopted from the causal inference literature, let exposure at baseline and follow-ups 1, 2, 3 be denoted by *A*_0_ , *A*_1_ , *A*_2_ , *A*_3_ and more generally, the history of exposure from baseline through time *t* be denoted ${\overset{-}{A}}_t={A}_0,\cdots, {A}_t$. The counterfactual outcome, for example at the final follow-up (time 4), when setting the exposure at follow-ups 1,2, and 3 to *A*_1_ = *a*_1_, *A*_2_ = *a*_2_, and *A*_3_ = *a*_3_ (small letters for realizations) is then denoted by ${Y}_{a_1,{a}_2,{a}_3}$. Generally, ${Y}_{{\overset{-}{a}}_{t-1}}(t)$ denotes the counterfactual outcome at time *t* for a specific hypothetical exposure history from baseline, through timepoint *t* − 1. In the simple univariate case, the MSM can take the form:

1 $$ E\left({Y}_{a_1,{a}_2,{a}_3}| X= x\right)=\alpha +{\beta}_0 x+{\beta}_1{a}_1+{\beta}_2{a}_2+{\beta}_3{a}_3 $$

where the average counterfactual outcome ${Y}_{a_1,{a}_2,{a}_3}$, conditional on baseline covariates (e.g. baseline exposure) is modeled as a linear function of the hypothetical levels of exposure at follow-ups 1, 2, and 3. The effect on the outcome of interventions on the exposure at follow-ups 1, 2, and 3 is *β*_1_, *β*_2_, and *β*_3_, for one unit changes. Each of these has a causal interpretation of a direct effect of an intervention at one point in time, on the outcome, conditional on the others (Fig. 1). With time – varying confounders affected by prior exposure, a standard regression model will in general give biased estimates of the exposure effect (Appendix). The MSM avoids this bias through confounder control by weighted regression, so called inverse-probability-of-treatment weighting, instead of including the confounders as covariates in eq. (1) as in standard regression (Appendix). To account for possible bias from differential loss to follow-up (censoring), inverse-probability-of-censoring weights were constructed in a manner similar to that described above for exposure (Appendix).

To assess the reciprocal association between each of the three personality functioning domains (adjusted for the other two) and psychosocial function (GAF), an MSM was fitted for the effects of hypothetical interventions on personality functioning (e.g., therapy) with GAF as outcome (one model for each SIPP domain). Then another MSM was fitted for the effects of hypothetical interventions on GAF (e.g., vocational rehabilitation) with SIPP as outcome (three separate models, one for each SIPP domain). The weights for all models included the following time-independent candidate confounders (*V* in Fig. 1 and Appendix): gender, age at baseline, years of education at baseline, medication one year prior to baseline (ranging from 0 indicating no medication to 3 indicating continuous medication for the past 12 months), marital status, living alone vs. not alone, and treatment condition (CP / OIP [6]). Time-varying confounders were past outcome and SIPP domains, possibly of multiple lags. For example, with REL as exposure and GAF as outcome, time-varying confounders consisted of prior REL, SLFC, IDENTITY and GAF. No variable was allowed to affect another at the same time-point (Fig. 1).

**Fig. 2:**
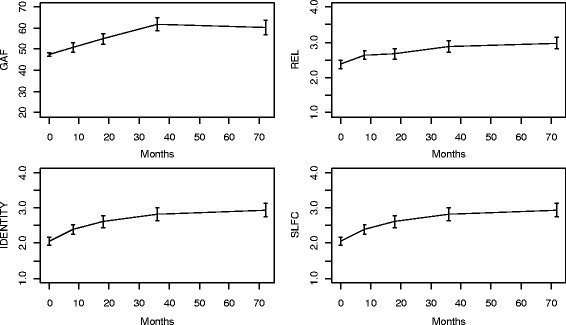
Psychosocial function (GAF) and three personality functioning domains REL, IDENTITY and SLFC in in a Norwegian sample of 113 patients with personality disorders, over six years of follow – up

In the analysis, hypothetical interventions on exposure at follow-up 1, did not have significant causal effects on the outcome at follow-up 4. By restricting the exposure history to two time-points prior to the outcome, a repeated measures MSM was fit, to better summarize and capture the dynamics in the data. The repeated-measures MSM simultaneously considers three different univariate models and averages over them: the effect of hypothetical interventions on exposure at baseline and follow-up 1 on outcome at follow-up 2, the effect of hypothetical interventions on exposure at follow-ups 1 and 2 on outcome at follow-up 3, and hypothetical interventions on exposure at follow-ups 2 and 3 on outcome at follow-up 4. Shorter intervals between follow-ups early in the study, as in the present design, are often used to achieve higher resolution where most changes occur, while limiting the number of follow-ups. With regards to SIPP domains and GAF, all four variables changed the most at the start of follow-up (Fig. 2) - a characteristic feature of treatment effects. Averaging over models with different lengths of intervals affects the interpretation of the repeated-measures MSM, with reference to increments of time (8 months between baseline and follow-up 1, versus three years between follow-ups 3 and 4). On the other hand, more similar changes are averaged over. The repeated-measures MSM with a non – linear term for change (supported in Fig. 1) was modeled in the following form:

2 $$ E\left({Y}_{{\overset{-}{a}}_{t-1}}(t)| X= x\right)={\alpha}_0+{\alpha}_1 t{+\alpha}_2{t}^2+{\beta}_0 x+{\beta}_1{a}_{t-1}+{\beta}_2{a}_{t-2} $$

with *X* denoting baseline exposure, and ${Y}_{{\overset{-}{a}}_{t-1}}(t)$ representing the outcome at follow-up *t* that would have resulted under hypothetical joint interventions to set exposure at follow-ups *t* − 1 and *t* − 2 to levels *a*_*t* − 1_ and *a*_*t* − 2_, respectively. In this model, the effects on the outcome at time *t* of joint interventions on the exposure at follow-ups *t* − 1 and *t* − 2 are *β*_1_ and *β*_2_, respectively, for a 1 – point change in exposure. Interactions between exposure at different time-points was assessed by including product terms. The weights for fitting this model vary over time and, at a particular time *t*, is the product of the weights up through time *t* − 1 [25]. To assess non-linearities in the regression models of the weights, general additive models (GAM) with splines were fitted for continuous variables [33].

To assess sensitivity for unmeasured confounding, the influence needed from a continuous unmeasured confounder to explain the observed associations, was calculated [34]. All analysis was performed using the statistical software R [35].

## Results

High reliability was found for all three SIPP domains at baseline, with alpha values of 0.88 for IDENTITY, 0.86 for SLFC and 0.86 for REL, and mean scores of 2.13 for IDENTITY (SD = 0.56), 2.52 for SLFC (SD = 0.64), and 2.41 for REL (SD = 0.69). Mean baseline, 8, 18, 36, and 72 months scores for GAF were 48.11 (SD = 5.14), 50.63 (SD = 10.13), 53.57 (SD = 10.75), 61.89 (SD = 13.37), and 62.54 (SD = 14.89). Large effect sizes were found for change from baseline to 72 months (*d* = (*μ*_72_ − *μ*_0_)/*σ*_*change*_) for both SIPP domains and GAF: *d* = 0.99 for GAF, *d* = 1.45 for IDENTITY, *d* = 1.26 for SLFC, and *d* = 1.35 for REL. These effect sizes indicated clinical improvement (Fig. 2).

### SIPP as exposure, GAF as outcome

Table 1 presents the results for the marginal effects over time of each of the three SIPP domains (IDENTITY, SLFC, and REL), adjusted for confounding, on GAF as outcome. The estimated coefficients describe the effects of hypothetical interventions on IDENTITY, SLFC, and REL at one and two time-points prior to GAF assessment. The results were non-significant for three time-points prior to GAF assessment, and for interactions between the different time-points.

**Table 1:** Estimated effects (regression coefficients) on psychosocial function – GAF of hypothetical interventions on the three personality functioning domains IDENTITY, SLFC and REL, one and two time – points prior to assessment of GAF in a Norwegian sample of 113 patients with personality disorders

	IDENTITY			SLFC			REL		
	Estimate	se	*p*-value	Estimate	se	*p*-value	Estimate	se	*p*-value
*t*	0.22	0.08	0.006	0.52	0.14	<0.001	1.92	0.38	<0.001
*t* ^2^	.	.	ns^a^	−0.004	0.002	0.02	−0.019	0.004	<0.001
*a*_*t* − 1_	6.58	1.73	<0.001	12.34	2.42	<0.001	16.1	3.33	<0.001
*a*_*t* − 2_	8.06	2.19	<0.001	.	.	ns^a^	8.3	4.09	0.042

^a^Non-significant terms were excluded from model

The results show differences between the personality functioning domains with regard to effect on psychosocial function. The model suggests that interventions to improve REL at both one and two time-points prior to GAF assessment would have an effect. A one-unit increase in REL at one time-point prior would increase GAF by 16.1 units (SE = 3.33; *p* < .001). Independent of this effect, an intervention to increase REL by one unit at two time-points prior would increase GAF by 8.3 units (SE = 4.09; *p* = .042). Coefficients for linear and non-linear time (months) were found to be significant (eq. 1). In other words, GAF would still increase (although at a slower rate) with hypothetical interventions to hold REL at a constant level at previous time-points (if such an intervention existed).

A hypothetical intervention that improved the IDENTITY score by one unit at one time-point prior to GAF assessment, would result in a GAF increase of 6.58 units (SE = 1.73; *p* < .001). Independent of this effect, an intervention improving the IDENTITY score by one unit at two time-points prior would give an additional GAF increase of 8.06 units (SE = 2.19; *p* < .001). The coefficient for linear time (in months) was found to be significant, interpreted as an increase in GAF even with hypothetical constant level of the IDENTITY score at previous time-points.

Finally, an intervention that improved SLFC by one unit at one time-point prior, would increase GAF by 12.34 units (SE = 2.42; *p* < .001). Coefficients for linear and non-linear time (months) were found to be significant, indicating increase in GAF even with hypothetical constant level of the SLFC score at previous time-points.

For the purpose of comparison, these effects can be expressed in terms of baseline standard deviations. A hypothetical intervention to increase REL by one standard deviation at one prior time-point would be expected to improve the GAF score by 2.3 standard deviations (95% CI: 1.37, 3.23). Intervening to improve REL by one standard deviation at two time-points prior would increase the GAF score by 1.19 standard deviations (95% CI: 0.04, 2.33). A hypothetical joint intervention to increase REL by one standard deviation, both at one and two time-points prior, would increase GAF score by 3.48 standard deviations (95% CI: 2.0, 4.96).

Similarly, an IDENTITY score increase of one standard deviation at one time-point prior would improve GAF by 0.88 standard deviations (95% CI: 0.43, 1.33), while an IDENTITY score increase of one standard deviation at two time-points prior, would increase GAF by 1.07 standard deviations (95% CI: 0.5, 1.65). Joint interventions to increase IDENTITY by one standard deviation at both prior time-points would increase GAF by a total of 1.95 standard deviations (95% CI: 1.22, 2.68). Furthermore, a hypothetical intervention that increased SLFC by one standard deviation at one time-point prior to assessment of GAF, would increase GAF by 1.71 standard deviations (95% CI: 1.05, 2.37).

**Fig. 3:**
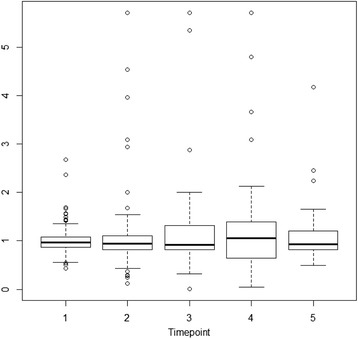
Boxplot of truncated (99th percentile) exposure weight distribution (SLFC) for each point of time 1 = baseline, …, 5 = 72 months) in a Norwegian sample of 113 patients with personality disorders

In accordance with assumptions, the distribution of the stabilized and truncated weights (for each time-point separately) had a mean close to one (Fig. 3). To examine the magnitude and direction of bias from time-varying confounding and censoring, the analyses were also performed without weights. Censoring appeared to have negligible effect. On the other hand, there was considerable time-varying confounding, mostly underestimation when confounder adjustment is dropped. For the REL domain, the effect of one time-point prior changed from 16.1 to 12.7, and the effect of two time-points prior changed from 8.3 to 2, indicating relative biases of 21% and 76%, respectively. For the IDENTITY domain, the effects changed from 6.58 and 8.06 to 8.7 and 4.9, respectively, indicating relative biases of −32% and 39%. For the SLFC domain, the effect changed from 12.34 to 6.6, indicating a 46% relative bias.

Feasible assessment of sensitivity for unmeasured confounding was achieved by splitting the repeated measures MSM into the three univariate MSMs (eq. 2, *t* = 2 , 3 , 4), for the association between GAF as outcome and REL at prior time-points as exposure. The regression coefficient for one time-point prior (strongest effect) was between 7.1 and 22.6. To assess the influence from a potential unmeasured confounder, necessary to fully explain the observed association with REL one time – point prior, intellectual ability (IQ) was thought of as a plausible candidate (for illustrational purpose). IQ has often been included as a covariate in models for various non-PD patient populations. In patients with bipolar disorder, a regression coefficient of 0.38 for IQ as an independent variable and GAF as dependent, has been reported [36]. In the present application this would translate to: An increase of one standard deviation in REL would have to correspond to an increase of 2 standard deviations in IQ to fully explain the observed univariate associations [34].

### GAF as exposure, SIPP as outcome

Table 2 presents results for the effects over time with psychosocial function (GAF) set as the exposure and personality functioning (IDENTITY, SLFC, and REL) as outcome— reversed models, compared to the above-described analysis. The estimated coefficients describe the effects of hypothetical interventions on GAF (e.g., vocational rehabilitation) at one and two time-points prior to SIPP assessment. As in the previously described analysis, effects for more than two time-points prior, and for interactions between different time-points were non-significant.

**Table 2:** Estimated effects (regression coefficients) on the three personality functioning domains IDENTITY, SLFC and REL of hypothetical interventions on psychosocial function – GAF, one and two time – points prior to assessment of personality functioning, in a Norwegian sample of 113 patients with personality disorders

	IDENTITY			SLFC			REL		
	Estimate	se	*p*-value	Estimate	se	*p*-value	Estimate	se	*p*-value
*t*	.	.	ns^a^	0.005	0.002	0.03	.	.	ns^a^
*GAF*_*t* − 1_	0.027	0.008	<0.001	0.023	0.005	<0.001	0.03	0.007	<0.001
*GAF*_*t* − 2_	.	.	ns^a^	.	.	ns^a^	0.035	0.011	0.002

^a^Non-significant terms were excluded from model

A hypothetical intervention that successfully improved GAF score by one unit at one time-point prior could be expected to increase REL by 0.03 units (SE = 0.007; *p* < .001), with adjustment for time-varying confounding from the other domains. Also, intervening to improve GAF at two time-points prior had an independent direct effect on REL of 0.035 units (SE = 0.011; *p* = .002). Coefficients for linear and non-linear time (in months) were found to be non-significant (eq. 2), i.e., REL would remain unchanged with hypothetical constant level of GAF at previous time-points.

A hypothetical intervention to improve GAF score by one unit at one time-point prior would increase IDENTITY by 0.027 units (SE = 0.008; *p* < .001). However, intervention at two time-points prior showed no direct effect. Coefficients for linear and non-linear time (in months) were again non-significant (eq. 2), i.e., IDENTITY would remain unchanged with hypothetical constant level of GAF at previous time-points.

Lastly, a hypothetical intervention to improve GAF score by one unit at one time-point prior would lead to a 0.023-unit increase of SLFC (SE = 0.005; *p* < .001). Again, intervention at two time-points prior showed no direct effect. A small coefficient for linear time was found, interpreted as a slight increase in SLFC, even with a hypothetical constant level of GAF at previous time-points.

For comparison, these results can be expressed in terms of baseline standard deviations.

A hypothetical intervention to increase GAF by one standard deviation at one time-point prior would be expected to improve REL by 0.21 standard deviations (95% CI: 0.11, 0.31). Intervening to improve GAF by one standard deviation at two time-points prior would increase REL by 0.24 standard deviations (95% CI: 0.09, 0.4). A hypothetical joint intervention to increase GAF by one standard deviation at both one and two time-points prior, would increase REL by 0.46 standard deviations (95% CI: 0.27, 0.64). A hypothetical increase of one standard deviation in GAF at one time-point prior would increase IDENTITY by 0.2 standard deviations (95% CI: 0.09, 0.32). Finally, a hypothetical GAF increase of one standard deviation at one time-point prior would increase the SLFC domain by 0.16 standard deviations (95% CI: 0.09, 0.24).

Again, censoring seemed to have negligible impact. Considerable time-varying confounding was observed when compared to results without weights, with both positive and negative bias. The mean of the stabilized weights was close to one (data not shown).

## Discussion

In the present study, a strong and reciprocal association between adaptive personality functioning and psychosocial function was found. These findings indicate that a successful intervention on one of the two constructs will also positively impact the other (causal effects). When focusing on how to improve one or both (or minimize deterioration), the feed-back mechanism facilitates further improvement via an additional pathway. The use of practical and cost-effective aspects could help optimize use of resources.

The presently applied causal model quantifies the different effects separately, estimating their persistence and accounting for time-varying (and time-fixed) confounding. While evidence showed effects in both directions between personality functioning and psychosocial function, the strongest effects were seen from personality functioning on psychosocial function (relative to baseline standard deviation). Within personality functioning, Relational Capacity was clearly the dominant domain. Partly overlap between items for this domain (e.g., “It is hard for me to feel loved by people close to me”) and the social function aspect of the GAF score gives some intuition for this finding.

Joint hypothetical interventions that could increase Relational Capacity by one standard deviation (0.6 units) at two succeeding time-points prior to GAF assessment, would be expected to lead to a GAF increase of 3.5 standard deviations (15.4 points), distributed as 2.3 for one time-point prior, and 1.2 standard deviations for two time-points prior. Given the mean baseline GAF value of 47.6, an increase of 15.4 points would result in an average GAF value of > 60, which is often regarded a clinical cut-off [3]. A GAF score of < 60 describes moderate-to-severe psychosocial dysfunction, while a GAF score of > 60 indicates mild dysfunction to high-level functioning. Thus, the present finding suggests that increasing Relational Capacity could induce substantial clinical improvement in psychosocial function. On the other hand, for the opposite causal effect, it was estimated that GAF improvements of one standard deviation at two succeeding time-points would lead to a total gain of 0.46 standard deviations in Relational Capacity (0.3 units), distributed as 0.21 and 0.24 standard deviations for the two preceding time-points. The difference in strength of effects between directions supports the view of personality functioning as one component of the more global construct of psychosocial function [7]. A change in psychosocial function affects many components, one of which is personality functioning, with further sub – categories of different domains. With regards to persistence of effects between Relational Capacity and GAF, evidence for long term influence in both directions was found, with significant coefficients for two prior time-points, representing a period of at least 18 months.

Compared to Relational Capacity, the other two domains of personality functioning—Self-control and Identity Integration—seemed to represent weaker causal mechanisms. Self-control at one time-point prior to GAF assessment had a strong effect on GAF, with short persistence (no significant effect from two time-points prior), also characteristic of the reverse effect. The effect of Identity Integration on GAF was of slightly smaller magnitude, but of longer persistence, with an additional effect from two time-points prior to GAF assessment. The reverse effect was of short persistence.

There are probably numerous reasons for the observed differences in length and (a)symmetry of persistence, between the different domains. In a factor – analysis, both Relational Capacity and Identity Integration loaded on a measure similar to GAF, whereas Self-control loaded on a different factor [15], in accordance with more long – term influence for more overlap. Identity Integration is closely associated with the patient’s ability to benefit from therapy [37] which seems to agree with the present finding that, of the three domains, Identity Integration had the highest effect size for change, also found elsewhere [37]. All three domains of personality functioning were found to be more temporal stable than GAF, with an average autocorrelation for different lags of 0.64 for Relational capacity, 0.62 for Self-control, 0.56 for Identity Integration and 0.3 for GAF. However, differential temporal stability does not seem to have contributed to asymmetric persistence for other than the Identity Integration domain.

The UPP study previously found that, within the subgroup of patients with borderline PD, the CP group achieved superior results from longitudinal analysis in Identity Integration and Self-control domains compared to the OIP group [38]. These findings are in line with results recently reported [19, 20]. A trend was found for Relational Capacity, but with a non-significant group × time interaction. One reason for weaker group difference in Relational Capacity might be that in the longitudinal model for each domain, no attempt was made to adjust for the others. Alternatively, the difference in treatment formats affected Relational Capacity to a lesser extent. Interestingly, patients in the CP group also showed larger increase in GAF during the 3- to 6-year follow-up period (post – treatment) compared to patients in the OIP group, consistent with causal influence from change in Identity Integration and Self-control to subsequent change in GAF in the borderline subgroup [38].

The results for the influence of time in the models (linear and non-linear) can be interpreted as support for both the improvement and lack of improvement in psychosocial function reported in the literature [3–6]. In the direction from personality functioning to psychosocial function, significant coefficients for time in all domains were found, suggesting increasing psychosocial function even when personality functioning is held constant. This agrees with the view of personality functioning as merely one component of psychosocial function, constituting a sufficient but not necessary contributor to improved psychosocial function. In the opposite direction, the lack of significant coefficients for time (in two of three domains) indicates that personality functioning remains unchanged when psychosocial function is held constant over time. Thus, both constructs serve as markers for each other—with improved personality functioning indicating improved psychosocial function, and persistent impairment in psychosocial function indicating persistent low personality functioning. Maladaptive personality functioning is one of many possible reasons for enduring impairment in psychosocial function. The present results indicate that if measured, personality functioning would show little improvement in cases where persistent impairment in psychosocial function has been reported, in contrast to the diagnostic remission over time [5].

**Fig. 4:**
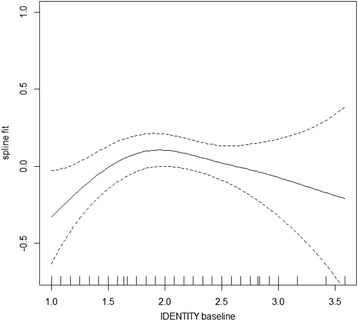
Non – linear association between IDENTITY at baseline and SLFC at 8 months (spline fit with 95% point-wise confidence interval), in a Norwegian sample of 113 patients with personality disorders

There is considerable discrepancy in estimated effects from the model presented here, compared to an ordinary repeated measures regression, like the linear mixed model (or the GEE estimator). Apart from an expected difference due to different effect measures (conditional versus marginal) this is due to time – varying confounding. Comparison of parameter estimates with and without weighting revealed considerable time-varying confounding, confirming the need to account for this bias. The direction of the bias was not as might have been expected, with positive associations between all time-varying variables. In the development of the personality functioning instrument (SIPP), the different domains were allowed to correlate (exploratory factor analysis with promax rotation) [17]. In the present application, the associations between domains were complex and non-monotone (Fig. 4). With four time-varying variables associated with and potentially affecting each other in complex ways, simple and intuitive rules for direction of confounding bias do not apply [39].

Measures of (mal)adaptive personality functioning, such as the Schedule for Nonadaptive and Adaptive Personality (SNAP), has been found to predict psychosocial function at 48 months later, but without a causal hypothesis or estimate of the strength of association [9]. A comprehensive dynamic longitudinal model recently confirmed prospective prediction of psychosocial function using DSM-5 personality traits within a structural equation model framework (SEM) [8]. Replications and extensions of these findings were called for. SEM models have a long tradition of use in psychology and represent an alternative to the MSM for causal inference, although they require more assumptions [23]. The present application represents an extension of the findings of Wright et al. to personality functioning, with a measure sensitive to mid- and long-term changes—which is also the temporal range for PD symptoms that have been clinically linked to psychosocial function [17, 40–42].

Adjustment for time-varying confounding yields separate marginal effect estimates for each domain of personality functioning. The causal interpretations of these effects are useful with respect to magnitude, persistence, and relative influence between different personality domains. However, there are several limitations. The sample size in this study is relatively large compared to other longitudinal studies of PDs, and with various PDs included, a strength of this study is generalizability to the PD population. However, from a statistical view, the sample size is a limitation. For regression models with a continuous response (like the inverse probability weights in the MSM), N/10 has been suggested as a maximum limit for number of covariates (with N as the sample size). With respect to this limit, the weights in the present analysis were more reliable at baseline (below limit) than at the end (above limit), due to a decrease in the sample size during the course of the study, from censoring*.* A causal interpretation relies on the untestable assumptions of “no unmeasured confounding” and “missing at random,” as well as “no model misspecification.” To assess the influence of violations to the “no unmeasured confounding” assumption, a simplified sensitivity analysis was performed. Splitting the repeated measures MSM into three univariate models showed that only very strong unmeasured confounding could “explain away” the observed associations between REL and GAF. As in many longitudinal studies, the “missing at random” assumption was probably not perfectly satisfied; however, selection bias from differential loss to follow-up was found to be negligible, suggesting small influence from this violation. In summary, based on effect magnitudes, the adjustment for a number of both baseline and time-varying confounders, and the sensitivity analysis results, it seems unlikely that the presently described effects were entirely due to unmeasured confounding. Interestingly, arguments for limited unmeasured confounding also include effects of treatment and support the causal pathway from personality functioning to psychosocial function (or vice versa). The limitation in the present data, of unmeasured treatment history on the individual level, illustrates this. Registered treatment history could enable estimation of the actual treatment effect, and e.g. how much of the treatment effect on psychosocial function that goes through personality functioning, so called mediation or indirect effect. With no mediation, a treatment effect on psychosocial function would come from a direct effect of treatment, and the treatment would represent a confounder between personality functioning and psychosocial function. There are several arguments in favor of an indirect effect. First, the three different domains of personality functioning, each had a strong association with psychosocial function, adjusted for the other two. This means that a direct potential treatment effect on psychosocial function, would have to account for the sum of the three separate associations, which is much larger than the observed change in psychosocial function. Second, the content of psychosocial function is wider than personality functioning, including for example symptoms and work – function, which were not intervened on in the treatment, and therefore represents an argument against a strong direct treatment effect. Third, the three domains are not all overlapping with psychosocial function [15], which can also be interpreted as an argument against an exclusive direct treatment effect on psychosocial function.

Improved knowledge regarding the association between personality and psychosocial function can help to reveal the nature and magnitude of the true causal mechanisms, and thereby contribute to improvement in psychosocial function for this patient group. A trial of randomized interventions targeting the specific personality domains and/or psychosocial function would bring us closer to the true effects. More observational studies, designed specifically to investigate this association and including precise therapy records and potential confounders, would also help to complete the picture.

## Conclusion

In conclusion, the present study indicates that persistent impairment in psychosocial function can be addressed through a causal pathway of personality functioning and strengthens the optimism for treatment of PD patients. Specifically, long-term interventions (at least 18 months), with emphasis on relational functioning and identity problems, and with long follow-up seem promising.

## Appendix

Biased estimation of exposure effect in a standard regression model is illustrated in Fig. 1. If the total effect of exposure at follow-ups 1, 2, and 3 on the final outcome is of interest, *L*_2_ should be adjusted for because it is a confounder for the effect of *A*_3_ on *Y*, but such an adjustment blocks the indirect effect of *A*_1_ on *Y* that passes through *L*_2_. In this way, bias might be generated whether or not *L*_2_ is adjusted for. The MSM avoids this bias through confounder control by weighted regression. Each individual is weighted by the inverse of his/her predicted probability for the observed exposure level, conditional on past covariate and outcome history. A weight is calculated for exposure at follow-ups 1, 2, and 3, and the overall exposure weight is the product. With continuous exposure, as in the present application, the probability is a conditional probability density obtained from a regression. In a similar manner, potential bias from differential loss to follow-up can be accounted for with inverse-probability-of-censoring weights. A series of logistic regressions are conducted (one for each time-point) to estimate the probability of not being lost to follow-up, and each individual is weighted with the inverse of this probability [22]. The total censoring weight is the cumulative product of the weights for each time-point, representing the probability of the observed censoring history for each person during the course of the study. Restrictions were made, that censored individuals were not allowed to re-enter the study at a later time. The final overall weight is the product of the exposure weight and the censoring weight.

In the repeated-measures MSM in eq. (2), the parameters were estimated by fitting a weighted conditional repeated-measures GEE model [43] with time-varying weights, to adjust for time-varying confounding and selection bias from monotone censoring. Robust standard errors were used due to the fact that the weights were estimated rather than known [22]. The conditional repeated-measures model is written as follows:

A.1 $$ E\left( Y(t)| X= x,{\overset{-}{A}}_{t-1}={\overset{-}{a}}_{t-1}\right)={\alpha}_0+{\alpha}_1 t{+\alpha}_2{t}^2+{\beta}_0 x+{\beta}_1{a}_{t-1}+{\beta}_2{a}_{t-2} $$

where *Y*(*t*) is the observed outcome at time-point *t*, and ${\overset{-}{a}}_{t-1}$ is the observed exposure history from baseline through time-point *t* − 1.

The weights comprised both exposure weights and censoring weights (both stabilized). The expression for the exposure weights is

A.2 $$ {SW}_A(t)={\prod}_{j=1}^t\frac{f\left({A}_j|{\overset{-}{A}}_{j-1}\right)}{f\left({A}_j| V,{\overset{-}{A}}_{j-1},{\overset{-}{L}}_{j-1},\overset{-}{Y}\left( j-1\right)\right)},\kern0.75em  t\ge 1 $$

where linear regression with normally distributed residuals are used to estimate the probability density function *f*(.). ${\overset{-}{L}}_{j-1}$ represents the history of the time-varying confounders up through time-point *j* − 1.

The censoring weights are given as

A.3 $$ {SW}_c(t)={\prod}_{j=1}^t\frac{\mathit{\Pr}\left({C}_j=0|{\overset{-}{C}}_{j-1}=0,{\overset{-}{A}}_{j-1}\right)}{\mathit{\Pr}\left({C}_j=0|{\overset{-}{C}}_{j-1}=0, V,{\overset{-}{A}}_{j-1},{\overset{-}{L}}_{j-1},\overset{-}{Y}\left( j-1\right)\right)} $$

where *C*_*j*_ = 0 is the dichotomous censoring indicator for remaining uncensored, and estimations are made through a series of logistic regressions.

The final time-varying weight is the product of the exposure and censoring weight, and is informally proportional to the probability of a person’s exposure and censoring history.

A.4 $$ {SW}_{tot}(t)={SW}_C(t){\times SW}_A(t) $$

To improve performance of the estimation, some recommendations from recent MSM – literature were followed. Precision was improved by using stabilized weights [22], and truncation of the weight distribution [44, 45]. The theoretical mean of one for the stabilized weights [46] can be used to test for indications of model miss-specification or violation of some underlying assumption for the MSM (e.g., the “positivity assumption”), which can be alleviated with truncation [44]. A recent review of MSM applications from 2000 to 2009 discussed presentation of results [47]. They found large differences in the magnitude of effect-estimates between conventional methods and MSMs, with infrequent reports of the mean of stabilized weights. In their longitudinal data, Cole and Hernan chose to separately examine and truncate the weights at each time-point [44]. Simulations have suggested that truncation at high percentiles is sufficient (here, the 99th percentile was chosen) in the right tail only, thus restricting maximum weights [45]. To reduce impact of misspecification, only variables considered as confounders and risk factors for the outcome were included in the weights, while pure predictors of exposure and censoring were excluded [48].

## Abbreviations

GAF: Global Assessment of Functioning
IDENTITY: Identity Integration
MSM: Marginal structural model
REL: Relational Capacity
SIPP: Severity Indices of Personality Problems
SLFC: Self-control
